# Prognostic Relevance of Estrogen Receptor Status in Circulating Tumor Cells in Breast Cancer Patients Treated With Endocrine Therapy

**DOI:** 10.3389/fonc.2022.866293

**Published:** 2022-04-28

**Authors:** Ying Zhou, Jinmei Zhou, Jinyi Xiao, Yuehua Wang, Hao Wang, Haoyuan Shi, Chunyan Yue, Fei Jia, Ping Li, Zhiyuan Hu, Yanlian Yang, Zefei Jiang, Tao Wang

**Affiliations:** ^1^Fujian Provincial Key Laboratory of Brain Aging and Neurodegenerative Diseases, School of Basic Medical Sciences, Fujian Medical University, Fuzhou, China; ^2^CAS Key Laboratory of Standardization and Measurement for Nanotechnology, CAS Key Laboratory for Biomedical Effects of Nanomaterials and Nanosafety, CAS Center for Excellence in Nanoscience, National Center for Nanoscience and Technology of China, Beijing, China; ^3^Breast Cancer Department, The Fifth Medical Center of PLA General Hospital, Beijing, China; ^4^School of Pharmaceutical Science and Technology, Health Science Platform, Tianjin University, Tianjin, China; ^5^Nanopep Biotech Co., Beijing, China; ^6^School of Nanoscience and Technology, Sino-Danish College, University of Chinese Academy of Sciences, Beijing, China; ^7^School of Chemical Engineering and Pharmacy, Wuhan Institute of Technology, Wuhan, China

**Keywords:** circulating tumor cells, breast cancer, estrogen receptor, liquid biopsy, prognostic

## Abstract

Recently, female breast cancer (BC) has surpassed lung cancer to occupy the first place of the most commonly diagnosed cancer. The unsatisfactory prognosis of endocrine therapy for breast cancer might be attributed to the discordance in estrogen receptor (ER) status between primary tumors and corresponding metastases, as well as temporal and spatial receptor status heterogeneity at point-in-time between biopsy and treatment. The purpose of this study was to evaluate the prognostic and predictive value of ER status in circulating tumor cells (CTCs) in BC patients. We analyzed ER expression on CTCs isolated using the Pep@MNPs method in 2.0 ml of blood samples from 70 patients with BC and 67 female controls. The predictive and prognostic value of ER expression in CTCs and immunohistochemistry results of biopsies for progression-free survival (PFS) and overall survival (OS) of patients in response to therapies were assessed. The detection rate for CTCs was 95.71% (67/70 patients), with a median of 8 CTCs within 2 ml of peripheral venous blood (PVB). A concordance of 76.56% in ER status between CTCs and corresponding primary tumor and 69.23% between CTCs and corresponding metastases was observed. We also found that patients with ER-positive CTCs (CTC ^ER+^) had longer PFS and OS than those without ER-positive CTCs (CTC ^ER-^). Our findings suggested that ER status in CTCs of BC patients may provide valuable predictive and prognostic insights into endocrine therapies, although further evaluation in larger prospective trials is required.

## Introduction

Recently, female breast cancer (BC) has surpassed lung cancer to occupy the first place of the most commonly diagnosed cancer, and it has been estimated that there were 2.3 million new cases worldwide in 2020 (1). In the era of precision medicine, the optimal therapeutic strategies in patient management depend on the determination of BC classification dividing into four molecular subtypes, luminal A, luminal B, human epidermal growth factor receptor 2 (HER2)-positive, and triple-negative breast cancer (TNBC), which are based on estrogen receptor (ER), progesterone receptor (PR), HER2, and Ki-67 expression (2). Notably, approximately 70% of BC patients are estimated to be ER-positive, and endocrine therapies have been the mainstay treatment for these patients (3–5).

ER status is considered one of the most pivotal predictive and prognostic factors in BC, but the level of ER expression may affect the prognosis of BC (6). Although adjuvant endocrine therapies after breast-conserving surgery significantly decrease the recurrence rates and improve the survival in patients with early-stage, ER-positive BC, the 5-year probability of BC recurrence is estimated to be around 20% and distant recurrences continue to occur for another 15 years (7, 8). Due to the rapid advancement of targeted therapy, the combination of cyclin-dependent kinases 4 and 6 (CDK4/6) inhibitors with first-line endocrine therapy has been used in late-stage development, but a large portion of patients may not benefit from the treatment (4, 9). Therefore, endocrine sensitivity among different BC patients has received considerable attention from clinicians for a long time. Correlations between ER expression and endocrine sensitivity have also been demonstrated (10, 11). The ER level between primary tumors and corresponding metastases is heterogeneous and shows dynamic changes during metastatic tumor progression (12–14). However, obtaining repeated biopsies in the metastatic site of late-stage BC patients is too difficult to determine the molecular characterization. Due to tumor heterogeneity and difficulty in sample biopsy for repeated analysis, the inaccuracy of single-site biopsies of the metastatic site may be detrimental to the determination of ER status and the optimization of treatment decisions. Therefore, in order to accurately and continuously assess ER status to monitor treatment response and disease progression, there is an urgent need for biomarkers with higher sensitivity and specificity.

Circulating tumor cells (CTCs) are recognized as minimally invasive, real-time liquid biomarkers due to the rare cell populations that are generated from primary or metastatic tumor cell clusters and flow into the blood, allowing early diagnosis during tumor progression and monitoring (15). The number of CTCs has been proved to be an independent predictor and a valuable prognostic marker for MBC patients’ progression-free survival (PFS) and overall survival (OS) (16–21). Nevertheless, whether CTCs would be of importance to predict the efficacy in response to endocrine therapy remains to be further investigated. Although ongoing research on CTCs focuses on mainly counting cells in peripheral venous blood (PVB), thorough research on the molecular characterization of CTCs might shed light on tumor progression and treatment resistance (22).

In recent years, discordance in the expression of ER between CTCs and primary tumors has been noticed and highlighted, and the alterations in ER status might be implicated in endocrine therapy resistance (23–25). However, many studies only focused on mRNA levels of ER instead of protein expression (26, 27). Studies determining ER status of each CTC isolated from patients are still limited. Inter-patient and intra-patient heterogeneity among CTCs ought to be further investigated. Whether ER status of CTCs could exert prognostic value remains controversial and more studies are urgently needed.

In our previous studies, we developed an efficient method using peptide-based iron oxide magnetic nanoparticles (Pep@MNPs) to enrich CTCs from PVB. Using 2.0 ml of PVB samples, the enumeration of CTCs by Pep@MNPs assays turned out to be clinically promising and had a predictive value for MBC patients (28–30). In this study, we aimed to characterize the ER status of isolated CTCs using the Pep@MNPs method and compared it to the ER expression profile of primary tumors and corresponding metastases. To determine the potential predictive and prognostic roles of ER status in CTCs in endocrine therapy, we analyzed the PFS and OS in diversified subgroups of patients.

## Materials and Methods

### Cell Lines and Cell Culture

Two human BC cell lines MCF-7 (CVCL_0031) (ER-positive) and MDA-MB-231 (CVCL_0062) (ER-negative) were purchased from Cell Bank of Chinese Academy of Sciences. Cells were cultured in Dulbecco’s Modified Eagle Medium (DMEM) (HyClone) supplemented with 10% (v/v) fetal bovine serum (FBS) (Gibco) and maintained at 37°C under 5% CO_2_ in a humidified incubator.

### Patients

For this study, a total of 70 BC patients from the Fifth Medical Center of PLA General Hospital were enrolled in this prospective open non-randomized study and their blood samples were collected from September 2017 to June 2018. Also, 67 female controls who volunteered to perform early screening for cancers were enrolled, and their blood samples were collected from January 2019 to July 2019 for CTC analysis. The study protocol was approved by the Ethics Committees of the Fifth Medical Center of PLA General Hospital. During the treatment period, some patients received different chemotherapies of metastatic settings, including anthracyclines, taxanes, capecitabine, and vinorelbine. Meanwhile, the others underwent endocrine treatment administrating drugs such as tamoxifen, aromatase inhibitors, and fulvestrant (**Supplementary Table S1**). The patients were divided into two groups (ER-positive patients and ER-negative patients) based on ER typing of primary tumor foci assayed by clinical immunohistochemistry (IHC) protocol. All patients and healthy volunteers had given written informed consent.

### Study Design

Eligible patients were required to have measurable or evaluable disease, with an Eastern Cooperative Oncology Group (ECOG) performance status score of 0 to 3, their ER and PR status, and the pathology report depicting their histological type and nodal status. PVB was drawn during a therapy cycle and delivered to the laboratory for CTC analysis. Only 2.0 ml of blood was used for CTC enumeration *via* Pep@MNPs method as described previously (28). CTCs were further analyzed for ER expression, and compared with primary tumor and metastases for ER status. The ER assessment of biopsies was determined by IHC in the Department of Pathology and ER is considered positive when at least 1% positive tumor nuclei exist in the sample as described (31). ER level was classified by an evaluation of the percentage of stained tumor cells and staining intensity. All treatment decisions for the patients were made according to the National Comprehensive Cancer Network (NCCN) clinical practice guidelines. The endocrine therapy selection was based on ER status in any one of primary tumors, metastases, or CTCs. Assessment of disease status was made according to Response Evaluation Criteria in Solid Tumors (RECIST) 1.1 criteria. After follow-up, the relationship between CTC ER expression and clinical outcome was evaluated by statistical analysis.

### Fluorescence-Activated Cell Sorting (FACS) and Immunofluorescence

To establish the fluorescent quantification system assessing ER expression by FACS and immunofluorescence, 1×10^6^ MCF-7 and MDA-MB-231 BC cells were digested with 0.25% trypsin, fixed in 4% paraformaldehyde for 15 min, and blocked with 5% BSA in PBST for 30 min. Subsequently, the cells were incubated with AlexaFluor 647-anti-ER antibody (Abcam, ab196159, 1:300) for 1 h at room temperature. After washing three times with PBS, the cells were incubated with secondary antibody for 1 h at room temperature. Nuclei were stained with 4’,6-diamidino-2-phenylindole (DAPI) (Abcam, ab285390, 1:2000). ER expression assessment was performed on the BD flow cytometer (BD Accuri™ C6), and the data were analyzed using Flowjo software (Tree Star). The staining results were obtained using a fluorescence microscope (OLYMPUS IX73). The immunofluorescent images were processed and measured by ImageJ software for quantification analysis.

### Isolation, Enumeration and Characterization of CTCs

CTCs were isolated and counted by the Pep@MNPs method as previously described (29, 32). In brief, the biotin-conjugated recognition peptide targeting epithelial cell adhesion molecule (EpCAM) with considerably high binding affinity was linked to streptavidin-conjugated iron oxide magnetic nanoparticles. For each detection, 10 μl pre-vortexed Pep@MNPs was added into 2.0 ml of the PVB sample, and incubated with gentle shaking at 37°C for 1 h. Subsequently, the captured CTCs were isolated under a magnetic field for 30 min and stained by multiple monoclonal antibodies (Abcam), including DAPI for cell nuclei, cytokeratin mix (Abcam, ab264485, 1:200) for positive selection, and CD45-phycoerythrin (Abcam, ab223183, 1:200) for negative selection (leukocytes). CTCs were identified as cells with the molecular feature of CKmix^+^/DAPI^+^/CD45^-^ under OLYMPUS IX73 fluorescence microscope. The expression of ER in CTCs was assessed with a Cy5-labeled anti-ER antibody (Abcam). Cell images were obtained and analyzed under OLYMPUS IX73 fluorescence microscope for final characterization of the magnetically captured cells.

### Statistical Analysis

All statistical computations were performed based on R version 3.6.3. The correlation between the number of CTCs that were detected in a sample and the percentage of ER-positive CTCs were analyzed using Spearman’s rank correlation coefficient. McNemar’s test was used to determine whether a statistically significant difference existed regarding variations in ER status between CTCs and histological results. P values <0.05 were considered statistically significant. P values <0.01 were considered extremely significant. Receiver operating characteristic (ROC) analyses were performed to evaluate the accordance of ER expression between CTCs and IHC results and implemented in python3.7.6. According to the proper threshold, samples were divided into two categories to compare median using the Wilcox test.

## Results

### Establishment of ER Quantitative Analysis Using Cell Lines and Patient Samples

**Figure 1:**
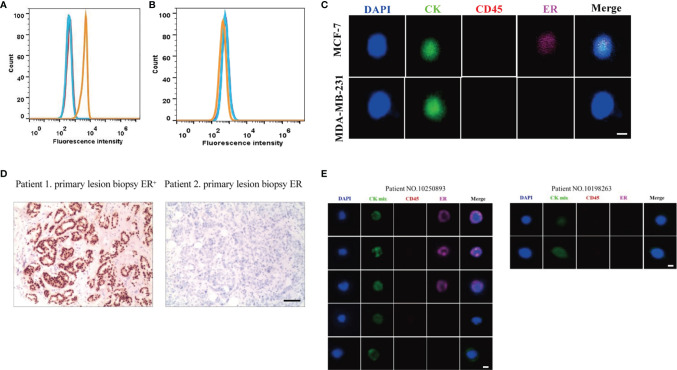
Establishment of ER quantitative analysis systems in BC cell lines and patient samples. **(A, B)** As shown by FACS, the fluorescence signal for ER was strong in MCF-7 BC cells **(A)**, but there was no significant signal in MDA-MB-231 BC cells **(B)**. Blue lines, unstained cells. Orange lines, stained cells. **(C)** MCF-7 BC cells were positive for ER, and MDA-MB-231 BC cells were negative for ER under a fluorescence microscope. **(D)** Based on the primary tumor IHC results, one patient was positive for ER while another patient was negative for ER. **(E)** Based on the Pep@MNPs method, one patient had both ER-positive CTCs (CTC^ER+^) and ER-negative CTCs (CTC^ER-^), while another patient only had ER-negative CTCs.

To assess the quantitative system for ER analysis, we first evaluated ER expressions in two extensively-used BC cell lines: MCF-7 and MDA-MB-231. Previous studies have shown that MCF-7 BC cells are ER-positive and low metastatic, whereas MDA-MB-231 BC cells are ER negative and highly metastatic (33). Our FACS results also showed that the fluorescence intensity of ER was 6.7×10^3^ in MCF-7 BC cells, 12.88 times higher than the unstained cells (**Figure 1A**). The fluorescence intensity was 4.8×10^2^ in MDA-MB-231 BC cells (**Figure 1B**), being similar with the unstained cells. Therefore, both BC cell lines were suitable for ER quantitative analysis.

Next, MCF-7 and MDA-MB-231 BC cells were isolated using the Pep@MNPs method and stained with Cy5-labeled antibodies against ER. In line with the FACS results, MCF-7 BC cells were positive for ER, while MDA-MB-231 BC cells were negative for ER under a fluorescence microscope (**Figure 1C**). The mean fluorescent intensity (MFI) of ER in each cell was calculated as described previously (32). Based on the MFI of isolated and stained cells, the threshold was set and defined an ER quantitative system: negative (ER^-^, MFI<150) and positive (ER^+^, MFI≥150).

Considering the importance of ER expression, clinical and histological characteristics of BC patients have been routinely assessed. After analysis of primary tumor samples from BC patients, we confirmed that one patient was positive for ER while another patient was negative for ER based on IHC assays (**Figure 1D**).

Using the previously established Pep@MNPs isolated system, CTCs characterized as CKmix^+^/DAPI^+^/CD45^-^ were captured from blood samples of BC patients and analyzed for ER expression. Specifically, the MFI of CKmix is equal to or greater than 120 and the MFI of CD45 is less than 80. As expected, in some patients (No. 10250893 as an example), CTCs being positive for ER (CTC^ER+^) were observed, while in other patients (No. 10198263 as an example) the results were negative (CTC^ER-^) (**Figure 1E**). Notably, among five CTCs isolated from the patient (No. 10250893), three CTCs were positive for ER, whereas two CTCs were negative for ER (**Figure 1E**), which presented a heterogeneity of ER expression in CTCs from an individual with BC.

### BC Patient and Control Group Characteristics

A summary of patient and tumor characteristics was listed in **Table 1**. A total of 70 BC patients enrolled whose blood samples were collected between September 2017 and June 2018 fulfilled the study criteria. The mean age of the patients was 51 years (range: 30 to 81 years). Among the 70 patients, 29 patients (41%) were in the early phase with a mean age of 52.5, while 41 patients (59%) were in the metastatic phase, with a mean age of 50 years. The available biopsies of a primary tumor and/or metastases in all patients were collected and analyzed. The biopsy analysis by IHC assays showed that a large proportion of patients were positive for ER. The percentage of patients with ER-positive primary tumor was 80% (56/70), while the percentage of patients with available metastases being ER-positive was 69% (20/29). 2.0 ml of the PVB samples was collected from each patient during the treatment to detect CTCs. We enrolled 67 females who volunteered to perform early screening for cancers, and their blood samples were collected for CTC analysis as controls to BC patients. The mean age of the control group was 49 years (range: 30 to 81 years), relatively following the characteristics of BC patients.

**Table 1:** Patient characteristics.

Feature	Number
Total patients	70
Age	
Median(Range)	51(30-81)
Disease phase	
EBC/Median age	29(41%)/52.5
MBC/Median age	41(59%)/50
Disease-free survival interval (months)	
Median(Range)	9(1-17)
Overall survival (months)	
Median(Range)	86(16-259)
ER-positive	
Primary lesion	80%(56/70)
Metastasis lesion	69%(20/29)
ER-negative	
Primary lesion	20%(14/70)
Metastasis lesion	31%(9/29)
HER2-positive	
Primary lesion	10%(7/70)
Metastasis lesion	13.8%(4/29)
PR-positive	
Primary lesion	65.7%(46/70)
Metastasis lesion	51.7%(15/29)
Metastasis site	
Visceral metastasis	98%(40/41)
non-visceralmetastasis	2%(1/41)

### Threshold of CTC Number Detected Between BC Patients and Healthy Controls

**Figure 2:**
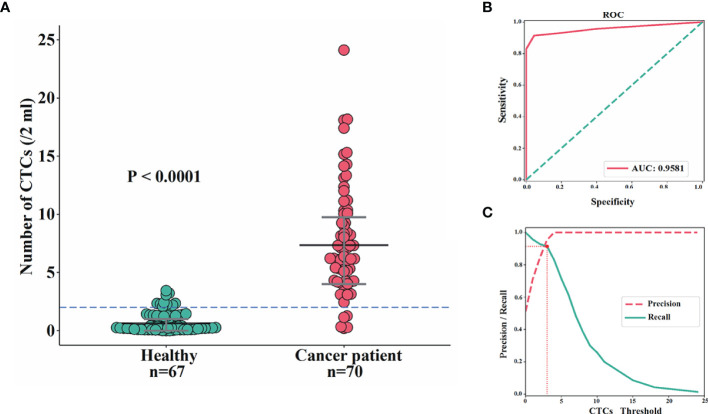
Comparisons of CTC enumeration between BC patients and female controls. **(A, B)** The difference of CTC numbers within 2 ml of blood between BC patients and female controls was extremely significant (P<0.0001) **(A)** with AUC being 95.81% **(B)**. **(C)** The optimal threshold based on precision-recall curve suggested that ≤2 CTCs within 2 ml of blood predict healthy females, whereas more than 2 CTCs predict patients.

In total, 70 blood samples from BC patients were collected and analyzed, and CTCs were detected in 67 cases. The detection rate for CTCs was 95.71%, which was consistent with our previous studies (32). The median number of CTCs in these patients was 8 (range: 1 to 24). By contrast, among 67 female controls, CTCs were detected in 27 volunteers, with the median number of CTCs being 2 (range: 1 to 3). The difference of CTC numbers within 2 ml of blood between BC patients and controls was extremely significant (P<0.0001), with area under the curve (AUC) being 95.81% (**Figures 2A, B**). The optimal threshold based on precision-recall curve suggested that ≤2 CTCs within 2 ml of blood predict healthy females, whereas more than 2 CTCs predict patients (**Figure 2C**). Therefore, the cut-off of >2 CTCs per 2 ml blood was applied for further analysis to evaluate that patients were considered CTC-positive.

### Assessment for Concordance in ER Status Between CTCs and the Corresponding Primary Tumor or Metastatic IHC Results

**Figure 3:**
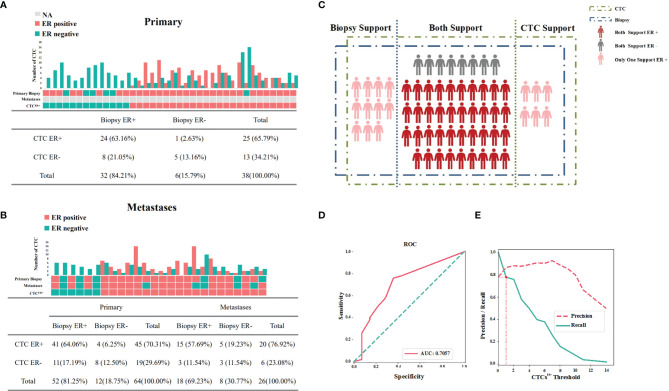
Expression profiles for ER of available IHC results and CTCs. **(A)** Concordance/discordance in the expression of ER between CTCs and corresponding primary tumors. **(B)** Concordance/discordance in the expression of ER between CTCs and corresponding metastatic IHC results. **(C)** Distribution of ER in CTCs and corresponding IHC results of the patients with available samples. **(D)** The area under the curve under the assumption that the accuracy of ER detection by IHC was 100%. **(E)** The optimal threshold on the basis of precision-recall curve suggested that at least one ER-positive CTC within 2 ml of blood be defined as CTC^ER+^.

To evaluate the correlation of ER expression between CTCs and the corresponding primary tumor, samples were analyzed from 38 patients whose primary tumor IHC results and CTCs were both available but metastatic information was not available. The number of CTCs, the number of ER-positive and ER-negative CTCs, the status of ER in primary tumor, and the status of ER in CTCs (CTC^ER+^≥1 designated as positive) were all evaluated in these patients. As illustrated in **Figure 3A**, 32 out of 38 (84.21%) patients were positive for ER in primary tumor, 25 out of 38 (65.79%) patients were positive for ER in CTCs, and 24 out of 38 (63.16%) patients were positive for ER both in primary tumor and CTCs. On the contrary, 5 out 38 (13.16%) patients were negative for ER both in primary tumors and CTCs. These data suggested that the concordance in ER status between CTCs and the corresponding primary tumor be 76.32%.

Then we further investigated the results from 26 MBC patients whose primary tumor IHC results, metastatic IHC results and CTCs were all available for assessment. As shown in **Figure 3B**, if these two subgroups with available results of primary tumor IHC and CTCs were both included, the concordance in ER status between CTCs and the corresponding primary tumor was 76.56% (49/64 cases). Among 26 MBC patients, metastases and CTCs displayed a concordant ER status in 69.23% (18/26) and a discordant ER status in 30.77% (8/26) of cases. Notably, discordance of ER expression between the primary tumor and metastases was found in four patients.

The ER detection results of 64 patients mentioned above were presented in **Figure 3C**, and the McNemar’s test demonstrated that the difference of ER detection rate between IHC results and CTCs in all cases was not significant (P=0.08). Assuming that the accuracy of ER detection by IHC was 100%, the AUC was calculated to be 70.57%, presenting a concordance to a certain degree (**Figure 3D**). Based on the precision-recall curve, the optimal threshold suggested that at least one ER-positive CTC within 2 ml of blood be defined as CTC^ER+^ (**Figure 3E**).

Taken together, these results indicated that there existed a relatively high concordance for ER status between CTCs and the corresponding primary tumor or metastatic IHC results, suggesting that ER expression in CTCs may be conducive to supplement the IHC results.

### Evaluation of Prognostic Responses of ER Status in CTCs and Corresponding IHC Results to Endocrine Therapy

To further compare the prognostic value of ER expression in CTCs and corresponding IHC results in response to endocrine therapy, we analyzed the data from patients whose primary tumor or metastatic IHC results, CTCs, and the first-line endocrine therapy efficacy when received CTC detection were available. The ER status and endocrine therapy efficacy in the subgroup of patients with available primary tumor IHC and CTC results (**Table 2**) and the subgroup of patients with available metastatic IHC and CTC results (**Table 3**) were listed. The highest disease control rate (DCR) was observed in patients whose IHC results and CTCs were positive for ER in both subgroups (79.2% and 86.7%, respectively). The second-highest DCR was shown in patients whose CTCs were positive for ER but IHC results were negative for ER in both subgroups (66.7% and 80.0%, respectively).

**Table 2:** Expression profiles for ER of IHC results and CTCs, and available PFS and DCR information in the subgroup of patients with available primary tumor IHC results.

Primary	Number	PFS (range/median)	Disease control rate (DCR%)
Biopsy ER^+^ & CTC ER^+^	24	1-7/4	79.2
Biopsy ER^+^ & CTC ER^-^	8	1-9/3	37.5
Biopsy ER^-^ & CTC ER^+^	3	2-17/5	66.7
Biopsy ER^-^ & CTC ER^-^	5	1-8/2	20

**Table 3:** Expression profiles for ER of IHC results and CTCs, and available PFS and DCR information in the subgroup of patients with available metastatic IHC results.

Metastases	Number	PFS (range/median)	Disease control rate (DCR%)
Biopsy ER^+^ & CTC ER^+^	15	1-7/5	86.7
Biopsy ER^+^ & CTC ER^-^	5	1-9/2	40
Biopsy ER^-^ & CTC ER^+^	5	1-17/5	80
Biopsy ER^-^ & CTC ER^-^	4	1-8/2	25

Next we focused on 10 patients who had a discordant ER status between the IHC results and CTCs (**Table 4**). Particularly, among 5 patients with ER-positive IHC results and ER-negative CTCs, 4 cases suggested poor results in response to endocrine therapy, while only 1 case was associated with favorable efficacy in response to endocrine therapy. For these patients, the accuracy of employing ER status in IHC results to instruct clinical treatment was only 20%. For 2 patients with ER-negative (primary tumor) IHC results and ER-positive CTCs, their responses to endocrine therapy were good, revealing that the accuracy of utilizing ER status in CTCs to predict clinical treatment be 100% for these patients. Among 5 patients with ER-negative (metastases) IHC results and ER-positive CTCs, 4 cases resulted in appreciative responses to endocrine therapy, demonstrating that the accuracy of applying ER status in CTCs to show clinical treatment was 80% for these patients. Therefore, the expression level of ER on CTC might better predict the efficacy of endocrine therapy when compared with IHC results.

**Table 4:** The statistics of 10 patients who had a discordant ER status between the IHC results and CTCs.

ID	Primary Biopsy-ER	Metastases Biopsy-ER	Number of single ER-positive CTCs	PFS	Efficacy
10171628	+	+	0	2	PD
10167720	+	+	0	2	PD
52078895	+	Unknown	0	2	PD
51498558	+	+	0	9	SD
52063558	+	Unknown	0	3	PD
52048167	+	–	14	1	PD
52118335	+	–	3	6	SD
52077287	+	–	6	5	SD
52078882	–	–	1	5	SD
10219372	–	–	4	17	SD

PD, progressive disease; SD, stable disease.

**Figure 4:**
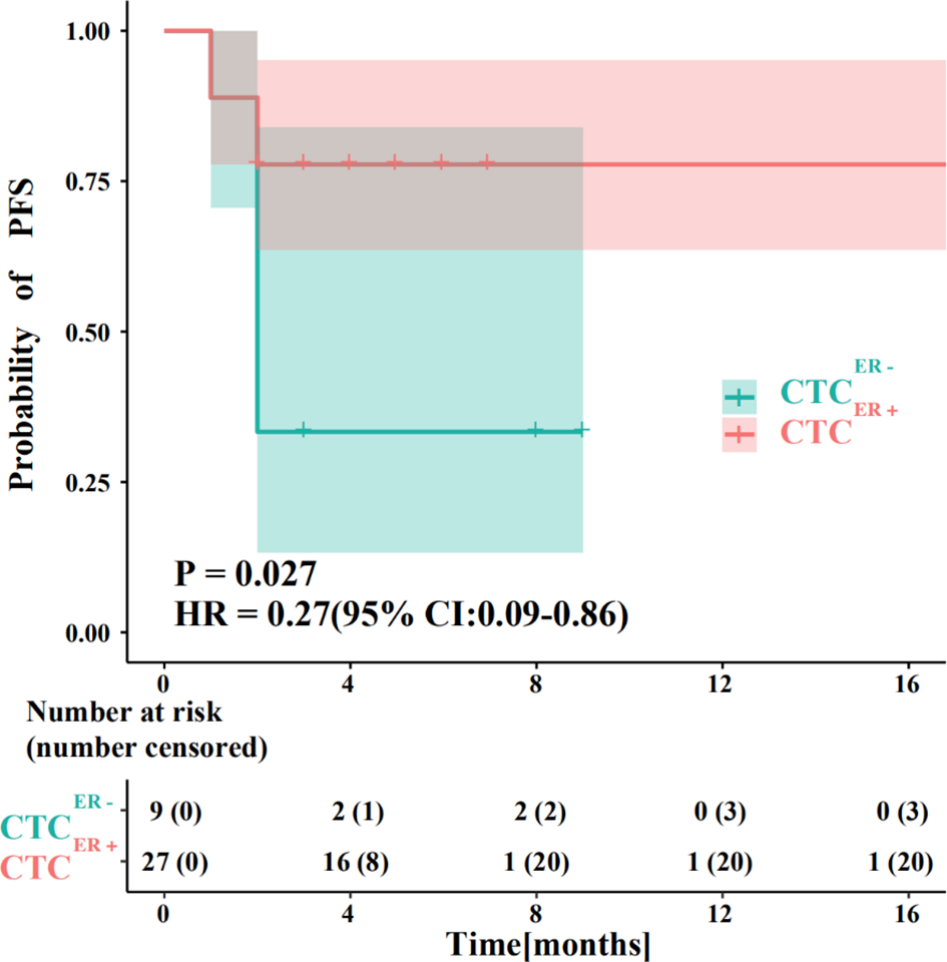
Evaluation of prognostic responses of ER status in CTCs and corresponding IHC results to endocrine therapy. The PFS between BC patients with ER-positive CTCs and those with ER-negative CTCs.

We also compared the PFS between patients with ER-positive CTCs and those with ER-negative CTCs. The results showed that a significant difference existed between these two groups (P<0.05), and the efficacies of endocrine therapy in patients with ER-positive CTCs was better than those of patients with ER-negative CTCs (**Figure 4**).

The advantage of ER detection in CTCs using our Pep@MNPs method was that we could conduct a multifaceted test that covered not only the determination of ER-positivity but also the acquisition of other metrics, including the numbers of total CTCs and ER-positive CTCs within 2 ml of PVB. In this study, we also found that ER-positive CTCs and ER-negative CTCs could coexist in the same patient, suggesting that the ratio of ER-positive CTCs to total CTCs (CTC^ER+^/CTC) may be relevant to the efficacies of endocrine therapy. When CTC^ER+^/CTC=0.25 was set as a critical value and all patients were divided into two groups accordingly, we found an obvious difference in PFS between the group of CTC^ER+^/CTC<0.25 and the group of CTC^ER+^/CTC≥0.25 (P=0.013) (**Supplementary Figure S1A**). Thus, we defined the patients of CTC^ER+^/CTC<0.25 as high-risk patients and the patients of CTC^ER+^/CTC≥0.25 as low-risk patients. Notably, in high-risk patients, the outcome of endocrine therapy was not effective, but in low-risk patients, the endocrine therapy had obvious curative effects. The PFS of high-risk patients was shorter than that of low-risk patients.

To sum up, our data showed that in diversified subgroups of patients with ER-positive CTCs (CTC ^ER+^) had longer PFS and better efficacy in response to endocrine therapy than those without ER-positive CTCs (CTC ^ER-^), demonstrating that ER status in CTCs might exert a predictive and prognostic significance whereas further evaluation was needed in larger prospective trials.

### Evaluation of ER Status in CTCs to Predict OS

**Figure 5:**
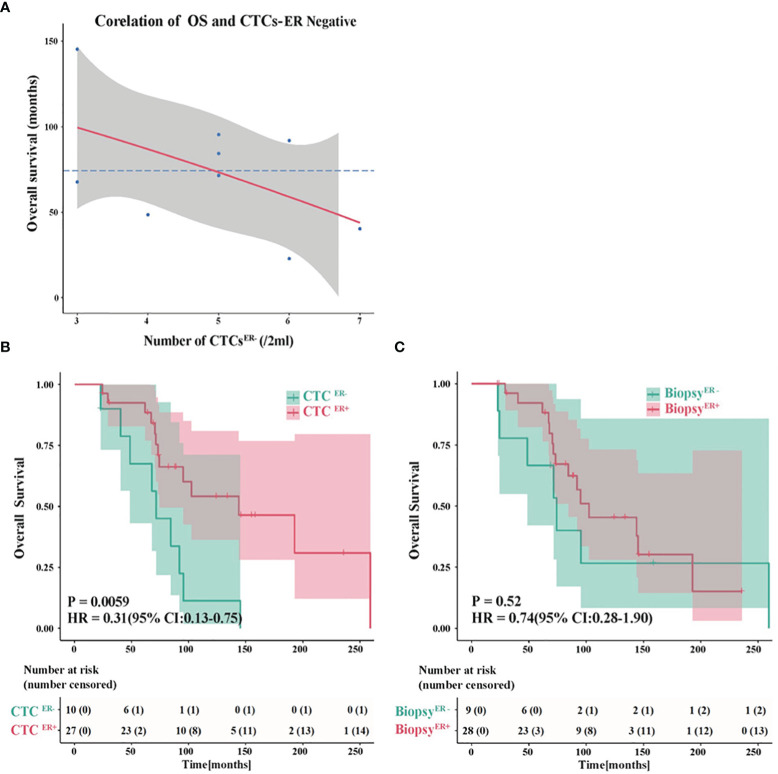
Evaluation of the ER status in CTCs to predict OS. **(A)** An inverse correlation between the number of CTC^ER-^ and OS in patients whose CTCs were negative for ER. **(B)** The probability of OS in patients with CTC^ER+^ was prominently different from that in CTC^ER-^ group (P<0.01). **(C)** The OS showed no significant difference between ER-positive and ER-negative patients based on the ER status of primary tumor or metastatic IHC results.

To further evaluate the potential roles of ER status in CTCs in predicting OS, we assessed the association between factors of interest and OS in BC patients. Interestingly, among patients whose CTCs were negative for ER, there was an inverse correlation between the number of CTC^ER-^ and OS (**Figure 5A**). The absence of ER in CTCs might compromise the efficacy of therapies, while the increasing number of ER-negative CTCs might facilitate metastasis and predict shorter OS.

Next, we compared the OS in patients with discordant status of ER in CTCs or IHC results. Remarkably, the probability of OS in patients with CTC^ER+^ was substantially different from that in CTC^ER-^ group (P<0.01, CTC^ER+^ median OS=143.9 months *vs* CTC^ER-^ median OS=71.5 months) (**Figure 5B**). Nevertheless, if the patients were grouped into ER-positive and ER-negative based on the ER status of primary tumor or metastatic IHC results, the OS showed no significant difference between these two groups (P=0.52) (**Figure 5C** and **Supplementary Figure S2**). These results suggested that the presence of CTC^ER+^ can predict patients with better OS outcomes. It is worth noting that between the group of CTC^ER+^/CTC<0.25 defined as high-risk and the group of CTC^ER+^/CTC≥0.25 defined as low-risk, the analytical results of OS were in accord with those of PFS (P<0.05) (**Supplementary Figure S1B**), indicating that the value of CTC^ER+^/CTC=0.25 was also applicable to predict OS.

## Discussion

According to the 8th edition of the American Joint Committee on Cancer (AJCC) Cancer Staging Manual, CTCs equal to or higher than 5 per 7.5 ml of PVB in advanced BC patients and CTCs equal to or higher than 1 per 7.5 ml of PVB in early phase BC patients both predict poor prognosis (34). The CellSearch^®^ system is the only laboratory test approved by the US Food and Drug Administration (FDA) for the enumeration of CTCs in MBC patients (35). Pep@MNPs method used in this study only used 2.0 ml of blood which needed a smaller amount of samples, but the detection rate was as high as 95.71%, which is consistent with our previous studies which also showed comparable capture efficiency (above 90%) using the highly sensitive method (28, 32). Nevertheless, a lower detection rate was observed in other studies, which might be attributed to assessment of RNA instead of protein or different detection method used (23, 24). Compared with the CellSearch^®^ system, the Pep@MNPs method might present elevated capture efficiencies (36). Among female volunteers, the median number of CTCs was 1 (range: 0 to 3). Compared with the controls, our results in BC patients presented a significant difference. In this study, CTC detection and IHC results were not exactly synchronized. The collection of blood samples exhibited varying degrees of latency so that some patients might have undergone specific treatments. We applied the cut-off of >2 CTCs per 2 ml blood for further analyzing the ER status, so available results from 64 patients were assessed. Therefore, the status of CTCs might dynamically changed corresponding to therapies, leading to discordant results between CTCs and IHC. On the other hand, this feature of CTC also makes it a more suitable tool for monitoring disease progression.

Recently, CTC clusters have been reported to be 20 to 100 times more metastatic than single CTCs in BC, and therapeutics targeting CTC clusters are being developed (37). However, compared with single CTCs, CTC clusters are much rarer in the circulation (38). Most of the CTC detection technologies are not developed specifically for CTC clusters, resulting in a possible inefficiency in the enrichment (37). In recent years, advanced technologies such as microchip and blood filtration have been developed, but requirement of specialized equipment and damage of CTC clusters may present limitations (39, 40). Therefore, more research is needed to develop and optimize the perfect approach for CTC cluster detection (37). Notably, in contrast to 7.5 ml of PVB used in the majority of these studies, we only collected and analyzed 2.0 ml of blood. It will be interesting in the future to evaluate CTC clusters within small amount of blood samples using our method.

Based on an evolving and prevailing hypothesis in recent years to explain tumor metastasis, the tumor cells in the central part and peripheral part of a solid tumor were fundamentally different regarding cellular functions (41, 42). These interior cells with high stemness maintained tranquility, while the surrounding cells presenting lower stemness possessed a strong penchant to migrate and invade. As the CTCs were shown to be closer to these surrounding cells highly correlated to metastasis, the sites for biopsies, however, were relatively randomized, which results in potential differences in the results. Since the interval between biopsies and treatment might be quite long, and errors might somehow exist in IHC results and CTC detection, further investigations were needed to determine which detection method would better guide the clinical treatment. In this study, there were 10 patients with intact IHC results and CTC data but showing discordant results. When the endocrine therapy matched CTC status, the DCR was 70% (7/10). When the endocrine therapy matched the IHC results of primary tumor, the DCR was 40% (4/10). When the endocrine therapy matched the IHC results of metastases, the DCR was 25% (2/8). Therefore, the expression levels of ER in CTCs may be conducive to supplement the IHC results to predict prognosis after endocrine therapy.

The molecular phenotype of tumor cells was dynamic and heterogeneous. It has been reported that 30-75% of patients who received surgeries and adjuvant therapies would suffer from recurrence resulting in MBC within approximately 2 years. Analysis of IHC results among MBC patients revealed that the detection rate of ER in metastases was lower than that in primary tumors. A theory proposed that after patients were first hospitalized and treated, the primary cause of the recurrence of patients, the temporary change in ER status in CTCs from positive to negative, which partly explained why tumor cells could be resistant to therapies after a period of hormone therapy or chemotherapy. Numerous MBC patients who suffered from recurrence might present the switch of a molecular phenotype (43). In this study, the comparative results of CTCs after treatment for the first time and CTCs after recurrence were not available. A thorough analysis of these data in further research might provide more evidence into clinical treatments. Interestingly, the ER status seemed to change back and forth among primary tumors, CTC and metastasis in some cases. In previous studies, it has been hypothesized that distant metastases development in BC patients with ER-positive primary tumors during or after endocrine therapy might be correlated with ER-negative CTCs (24). ER expression could be modulated by both genomic and non-genomic pathways (44). ER loss might be induced by overactivation of growth factor receptor pathways (45). Notably, some ER-negative tumors have been observed to become ER-positive and the patients can subsequently benefit from the endocrine therapy (46). If confirmed, the switch of ER expression might provide insights into the cause of resistance to endocrine therapy, and determination of ER status of CTCs might hold a promising prognostic significance.

The existence of an ER-/PR+ subgroup remains controversial, and is sometimes regarded as the result of technical artifact (47). However, other studies have shown the clinical and biological significance of ER-/PR+ as a distinct phenotype (48–53). The ER-/PR+ patients also received endocrine therapy (48, 49, 54). Notably, it has been reported that adjuvant tamoxifen therapy could increase the survival benefit in terms of OS and disease-free survival in patients with low-grade ER-/PR+ tumors (55). Due to the randomized collection of samples and limitation of detection methods, both IHC and CTC detection results might present a misdiagnosis rate (56). Therefore, the therapeutic efficacy in patients needed to be combined to assess the accuracy of detection technologies. In this study, we combined the PFS and OS results of patients to evaluate the predictive value of both IHC and CTC detection results in clinical endocrine therapy. The data suggested that the detection of ER status in CTCs in combination of the results of IHC might better guide clinical treatment. For a patient with ER-positive IHC results, both ER-positive CTCs and ER-negative CTCs existed. However, the percentage of these specific CTCs could, to some extent, reflect the condition in response to endocrine therapy. As the blood samples might be collected from patients at any time point before, in the middle, or after the therapy, the treatment tended to have dual effects on the number of both total CTCs and ER-positive CTCs. Since both results changed simultaneously in response to treatment, the ratio of ER-positive CTCs to total CTCs was taken into account for the evaluation. Based on our analysis, this characterization method turned out to be effective. High-risk patients and low-risk patients split accordingly, and the PFS and OS between these two subgroups presented striking differences, which might guide treatment more precisely.

Through the statistical analysis of the number of CTCs and OS, we found that among patients with CTC^ER-^, between the number of CTCs and OS was a negative correlation. This result agreed with previous reports that an increasing number of CTCs in patients predicted a shorter OS (57). Yet among patients with CTC^ER+^, no significant correlation was presented between the number of CTCs and OS (data not shown). A possible explanation was that patients with ER-positive CTCs might have better efficacy in response to endocrine therapy. Even though they had a larger number of CTCs, they might also have a better prognosis, which rationalized a departure from this negative correlation.

In conclusion, we demonstrate here the prospective evaluation of the status of ER expression in CTCs taking advantages of an efficient peptide-based CTC isolation system used to verify their predictive and prognostic value of endocrine therapy in patients with BC. Compared with traditional determination of ER status through IHC, assessment results of ER status in CTCs of BC patients may provide valuable independent predictive and prognostic information for endocrine therapies, which proves that liquid biopsy may be used to stratify for benefits of endocrine therapy among patients with BC and guide therapy.

## Data Availability Statement

The original contributions presented in the study are included in the article/**Supplementary Material**. Further inquiries can be directed to the corresponding authors.

## Ethics Statement

The studies involving human participants were reviewed and approved by the Ethics Committees of the Fifth Medical Center of PLA General Hospital. The patients/participants provided their written informed consent to participate in this study.

## Author Contributions

ZH, YY, ZJ, and TW contributed to conception and design. YY, HW, JZ, and CY contributed to development of methodology. JX, YW, FJ, and PL contributed to data collection. YZ, JZ and HS contributed to data analysis and interpretation. YZ, ZH, ZJ, and TW contributed to manuscript writing. All authors contributed to review and approval of the manuscript.

## Funding

This study was supported by grants from Research Foundation for Advanced Talents of Fujian Medical University (XRCZX2017020, XRCZX2019005), Beijing Natural Science Foundation (7192198), Strategic Priority Research Program of Chinese Academy of Sciences (XDB36000000, XDB38010400), National Natural Science Foundation of China (32027801, 31870992, 21775031), CAS-JSPS (GJHZ2094), Science and Technology Service Network Initiative of the Chinese Academy of Sciences (KFJ-STS-ZDTP-079).

## Conflict of Interest

Author HW was employed by Nanopep Biotech Co.

The remaining authors declare that the research was conducted in the absence of any commercial or financial relationships that could be construed as a potential conflict of interest.

## Publisher’s Note

All claims expressed in this article are solely those of the authors and do not necessarily represent those of their affiliated organizations, or those of the publisher, the editors and the reviewers. Any product that may be evaluated in this article, or claim that may be made by its manufacturer, is not guaranteed or endorsed by the publisher.
