# Supplementary material for: Prognostic Relevance of Estrogen Receptor Status in Circulating Tumor Cells in Breast Cancer Patients Treated With Endocrine Therapy
Source: Front Oncol. 2022 Apr 28;12:866293. doi: 10.3389/fonc.2022.866293 (PMC9097586; doi:10.3389/fonc.2022.866293)
Supplement: Supplementary file 1 [file DataSheet_1.pdf]

Supplementary Material

1 Supplementary Figures and Tables

1.1 Supplementary Table

Supplementary Table 1. Characterization of endocrine therapies included in the study.

Category based on mechanisms	Drugs	Number (percentage)	Disease control rate (DCR%)	PFS (range/ median)
Aromatase inhibitor (AI)	Exemestane	8 (22.2%)	62.5	1-5/3
Aromatase inhibitor (AI)	Anastrozole	8 (22.2%)	62.5	2-9/4.5
Aromatase inhibitor (AI)	Letrozole	1 (2.8%)	100	6/6
Selective estrogen receptor modulator (SERM)	Toremifene	2 (5.6%)	50	2-5/3.5
Selective estrogen receptor modulator (SERM)	Tamoxifen	4 (11.1%)	100	6-9/7
Selective estrogen receptor modulator (SERM)	Fulvestrant	10 (27.8%)	60	1-7/3.5
Progestogen	Medroxyproges terone	3 (8.3%)	66.7	1-17/3

1.2 Supplementary Figures

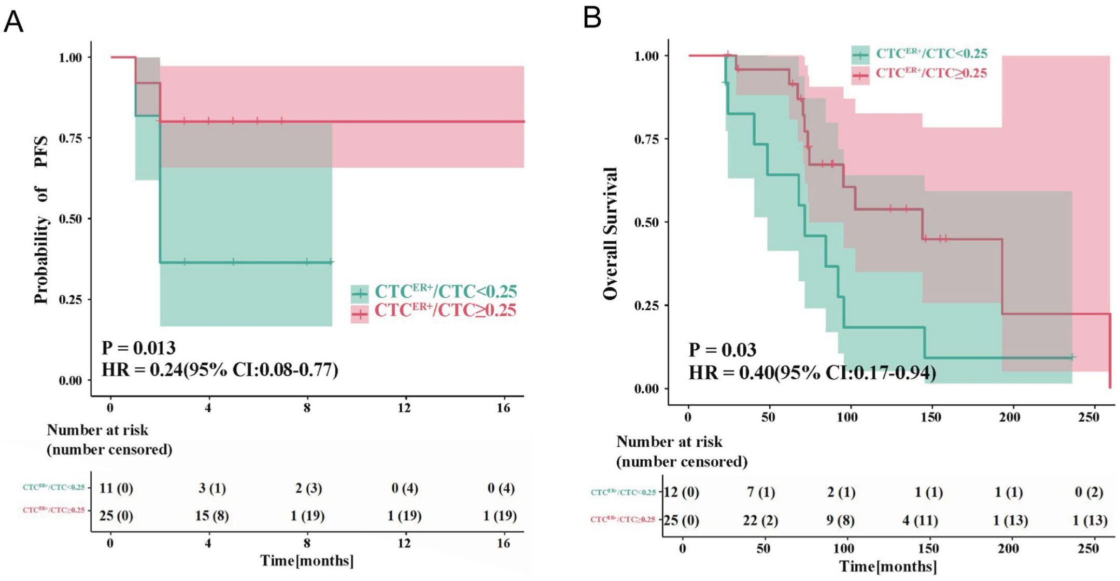

Supplementary Figure 1. Evaluation of the ratio of ER-positive CTCs to total CTCs in response to endocrine therapy to predict PFS and OS. **(A)** The PFS between the group of $CTC^{ER+}/CTC<0.25$ and the group of $CTC^{ER+}/CTC\geq0.25$ showed that there existed a significant difference between these two groups ($P<0.05$). **(B)** The OS between the group of $CTC^{ER+}/CTC<0.25$ and the group of $CTC^{ER+}/CTC\geq0.25$ showed that there existed a significant difference between these two groups ($P<0.05$).

1.3 Supplementary Figures

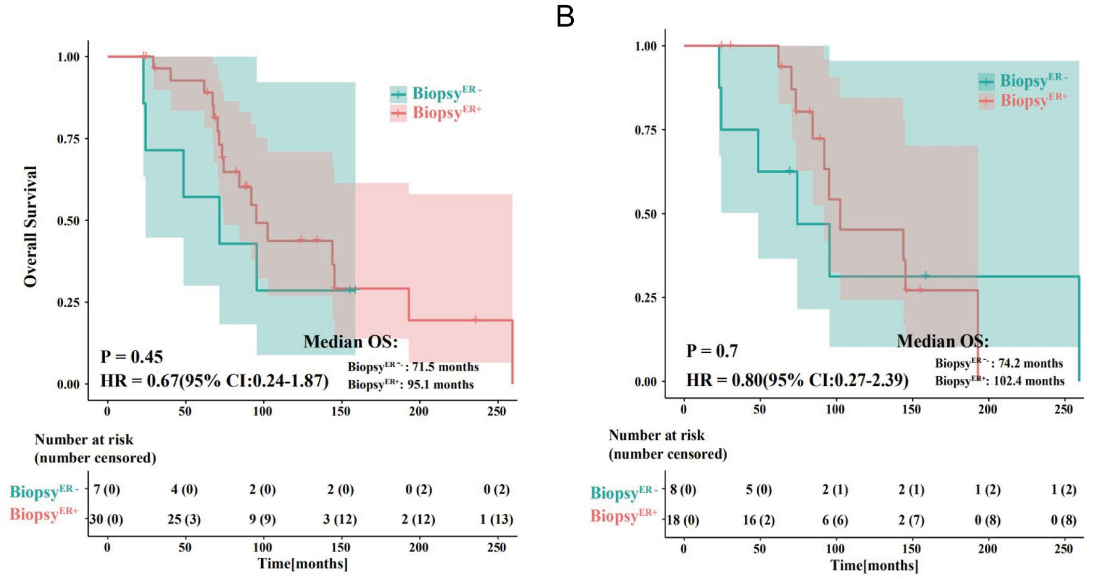

Supplementary Figure 2. Evaluation of ER expression in primary lesion and metastasis lesion IHC results to predict OS. **(A)** While the patients on the basis of the ER status of the primary lesion IHC results into ER-positive and ER-negative, the OS showed no significant difference between these two groups (P=0.45). **(B)** While the patients on the basis of the ER status of the metastasis lesion IHC results into ER-positive and ER-negative, the OS showed no significant difference between these two groups (P=0.7).
